# Supplementary material for: Gut microbiota of Brazilian Melipona stingless bees: dominant members and their localization in different gut regions
Source: bioRxiv. 2025 Jun 4:2025.06.03.657762. Preprint. [Version 1] doi: 10.1101/2025.06.03.657762 (PMC12157432; doi:10.1101/2025.06.03.657762)
Supplement: 1 [file NIHPP2025.06.03.657762v1-supplement-1.pdf]

bioRxiv preprint doi: https://doi.org/10.1101/2025.06.03.657762; this version posted June 4, 2025. The copyright holder for this preprint (which was not certified by peer review) is the author/funder, who has granted bioRxiv a license to display the preprint in perpetuity. It is made available under aCC-BY 4.0 International license.

Supporting information

S1 File. Supporting tables and figures. This PDF contains (1) S1 Table. Information of collection, species name and source of the *Melipona* samples analyzed in the present work. (2) S2 Table. PERMANOVA based on the Bray-Curtis dissimilarity matrix comparing the differences in the microbial community composition between the gut regions of *M. quadrifasciata*. (3) S3 Table. GenBank sequences used for analysis. (4) S1 Figure. NMDS plot based on ASV relative abundance using a Bray-Curtis dissimilarity matrix, illustrating bacterial community composition across different *Melipona* species and biomes. Colors represent bee species, with color groupings indicating *Melipona* subgenera: orange – *Melipona*, green – *Michmelia*, blue – *Eomelipona*, and pink – *Melikerria*. Point shapes denote the biome of origin. (5) S2 Figure. Most abundant families in *Melipona* spp. gut microbiota. Each sample represents a pool of 5 bees per box per site of study. ASVs are ordered and colored at the family level, with low abundant ASVs grouped as 'Other'. (6) S3 Figure. Most abundant genera in *Melipona* spp. gut microbiota. Each sample represents a pool of 5 bees per box per site of study. ASVs are ordered and colored at the genus level, with low abundant ASVs grouped as 'Other'. (7) S4 Figure. Bacterial alpha diversity of the gut regions of *M. quadrifasciata*. The alpha diversity was expressed using the Shannon and richness indexes. A Kruskal-Wallis test ($p < 0.05$) was conducted, followed by a post-hoc pairwise Dunn test to compare each gut part, showing only the significant results. (8) S5 Figure. Phylogenetic trees of the most abundant ASVs (including the 11 core ASVs) found in *Melipona* bee populations. Bootstrap values are shown in blue letters. The 11 core ASVs are written in bold characters. ^{T} Type strain. Trees are shown for the most abundant and core ASVs of A) *Apilactobacillus*, B) *Lactobacillus*, C) Streptococcaceae, D) Bifidobacteriaceae, and E) Acetobacteraceae. The phylogenetic trees were rooted according to the outgroups: (A) *Fructilactobacillus fructivorans*, (B) *Amylolactobacillus amylophilus,* (C) *Lactiplantibacillus plantarum*, (D) *Bombiscardovia coagulans*, (E) *Granulibacter bethesdensis.*
